# Analysis of breast milk fatty acid composition using dried milk samples

**DOI:** 10.1186/s13006-016-0060-2

**Published:** 2016-01-25

**Authors:** Kristina Harris Jackson, Jason Polreis, Laura Sanborn, David Chaima, William S. Harris

**Affiliations:** OmegaQuant Analytics LLC, Sioux Falls, SD USA; Department of Microbiology, University of Malawi, College of Medicine, Blantyre, Malawi; Sanford School of Medicine, University of South Dakota, Sioux Falls, SD USA

**Keywords:** Breast milk, Docosahexaenoic acid, Assessment of nutritional status, Lactation, Infant and child nutrition, Maternal nutrition

## Abstract

**Background:**

The effect of breast milk fatty acid (FA) composition, particularly levels of docosahexaenoic acid (DHA), on infant health outcomes is unclear. Part of the reason for this is difficulties in collecting, storing and shipping milk samples to the laboratory. Here we report the validation of a dried milk spot (DMS) system to measure FA composition to help overcome these obstacles.

Milk FA were measured by gas chromatography and reported as percent of total FA; the FA of primary interest in this study were DHA and industrially produced *trans* FA (iTFA). Experiments were carried out using pooled milk samples from US (*n* = 5) and Malawian women (*n* = 50). Experiments compared liquid vs. DMS samples (*n* = 55), assessed stability of FA composition under different storage conditions (*n* = 5), and compared the results from two different labs using the same methods (*n* = 5).

**Results:**

Both % DHA and % iTFA levels in liquid and DMS samples were strongly correlated (R^2^ = 0.99 and 0.99, respectively, *P* < 0.0001). The % DHA in DMS samples was stable for up to four weeks at room temperature and up to three years at -80 °C; only slight deviations from the acceptable range of variability (±15 %) occurred in the 4 °C and -20 °C conditions for % DHA. The % iTFA was stable under all conditions. All % DHA and % iTFA were within 15 % of the referent when analyzed in two laboratories.

**Conclusions:**

Valid FA composition values can be obtained from DMS samples using this robust collection and transport system which should facilitate studies of the role of milk FA composition in infant development.

**Electronic supplementary material:**

The online version of this article (doi:10.1186/s13006-016-0060-2) contains supplementary material, which is available to authorized users.

## Background

The effect of breast milk fatty acid (FA) composition on infant health outcomes is a promising area of research. Higher docosahexaenoic acid (DHA, C22:6n3) levels in milk have been linked to improved visual or neurological outcomes in term infants in several (but not all) studies [1, 2]. Milk DHA levels vary widely around the world [3] and are highly correlated to the mother’s intake of DHA-rich foods [4] or DHA/fish oil supplement use [5]. Similar to DHA, industrial *trans* FA levels (iTFA; C18:1 *t*, C18:2n6*t*) in milk are highly related to the mother’s intake of foods containing *trans* fats, i.e. some processed foods and meats, because the body does not synthesize them [6]. Milk iTFAs were found to be inversely related to long-chain polyunsaturated FA levels, including DHA, in a cohort of German women [6]. iTFA has been shown to block the synthesis of DHA from its precursor, alpha-linolenic acid [7, 8]. Because DHA and iTFA levels in milk are largely diet-dependent (unlike most other milk FA), there is an opportunity for nutritional interventions to alter milk FA profile and determine the effects on infant outcomes. Research in this area, particularly in under-developed regions, has been hampered by difficulties in collecting, storing and shipping milk samples to the laboratory for analysis. Dried *blood* spots on antioxidant-treated filter paper have proven to greatly reduce logistical issues and subject burden during collection while providing a reliable sample for FA analysis [9, 10]. Here we report on the validation of a dried *milk* spot (DMS) system for determining milk FA composition that can help overcome some of these same obstacles.

## Methods

### Sources of milk samples used in validation experiments

Pooled samples of liquid milk from US lactating women (*n* = 5) with a relatively wide range of DHA levels (0.05 to 0.42 % DHA) that had been submitted for routine FA analysis were de-identified and used for method development. After the initial analysis, samples were stored at -80 °C until stability and validity experiments were conducted.

A second set of samples was collected in Malawi, Africa (*n* = 50) as part of a randomized trial of a lipid-based nutritive supplement (which did not include DHA or iTFA) for pregnant women [11]. The trial was performed according to Good Clinical Practice guidelines and the ethical standards of the Helsinki Declaration. The protocol was approved by the College of Medicine Research and Ethics Committee, University of Malawi and the Ethics Committee of Pirkanmaa Hospital District, Finland. This trial was registered at clinicaltrials.gov with the identifier NCT01239693.

Breast milk samples from Malawi were collected at participants’ homes by following milk expression instructions that were given by female sample and data collectors. The mothers expressed the milk into clean plastic cups, which was immediately mixed thoroughly with a clean plastic spoon. Ten milliliters of milk was collected from this mixture into a clean falcon tube (the rest was given back for the child) and placed on ice for at most one hour. This was then taken to a satellite laboratory and aliquoted into five, 2 ml cryovials. These were stored temporarily at the satellite laboratory at -20 °C for no more than 2 days after which they were taken to a central sample archive laboratory and placed at -80 °C for long-term storage. The samples were shipped to the US on dry ice in accordance with International Air Transport Association sample shipment instructions.

### Preparation of dried milk spots (DMS)

All DMS samples were prepared in the laboratory by placing two drops (≈ 50 uL) of thawed milk on absorbent paper (Ahlstrom 226, PerkinElmer, Greenville, SC) pretreated with OxyStop®, a proprietary antioxidant cocktail which delays oxidation of FA in dried whole blood [12].

### Laboratory methods

A hole punch from the DMS card or 12.5 uL of thawed liquid milk were combined (1:40 parts) with the methylating mixture [boron trifluoride in methanol (14 %), toluene and methanol (35/30/35 v/v)], shaken and heated at 100 °C for 45 min. After cooling, 40 parts of both hexane and distilled water were added. After briefly vortexing the samples were spun to separate layers, and an aliquot of the hexane (upper) layer which contained the FA methyl esters was taken for analysis by gas chromatography (Shimadzu 2010; SP2560, 100-m column) as described previously [12]. Data are expressed as a percent of total identified FA; a total of 26 FA between C10:0 – C22:6n-3 were identified. With each batch of DMS samples analyzed, two controls (one high and one low in DHA) were run. A 3-point standard curve was run at the beginning of each batch from which DHA levels of the unknowns were determined. Pure standards had been used to confirm the identities of DHA and iTFA, and additional confirmation has been made using gas chromatography-mass spectroscopy in our method development. The CVs for DHA were 6.0 % and iTFA 3.8 %.

### Validation of liquid vs. dried milk spot fatty acid profile

Fifty-five samples of milk from Malawian and US mothers were used to compare the FA composition of liquid vs. DMS. To make DMS samples, 1iquid milk samples were thawed and one drop of milk was placed on the collection cards. Then the DMS samples were stored at room temperature in the dark for one week before analysis (to simulate the approximate time from collection to analysis in the field). The liquid samples analyzed immediately after thawing.

### Stability of fatty acid profile in dried milk spots: varying time and temperature

The five pooled milk samples from US mothers were spotted onto pretreated cards. Several DMS cards from each pool were stored in the dark in reclosable plastic bags under each of the following conditions: room temperature (23 °C), refrigeration (4 °C), standard freezer (-20 °C), and research freezer (-80 °C). At Days 4, 7, 14, 21, and 28, DMS from the first three storage temperatures were analyzed for FA composition; the Day 0 sample was analyzed immediately after spotting and served as a reference. Additionally, DMS stored at -20 °C and -80 °C were tested at months 3, 6, 9, and 12, and then every 6 months for up to 3 years.

### Interlab validation

Pooled milk samples from five US mothers were placed on DMS cards and sent by express mail at ambient temperature to an affiliated lab in Seoul, South Korea (OmegaQuant Asia). Identical DMS samples were saved at OmegaQuant Analytics in Sioux Falls, SD. Sample analysis was coordinated to be done on the same day (about 1 week after shipping). Identical methods were used in both laboratories.

### Statistical methods

All statistics and graphs were analyzed and created with MiniTab (Student version, State College, PA, USA) and GraphPad Prism 6 (GraphPad software, San Diego, CA, USA). The primary FA of interest were DHA (C22:6n-3) and iTFA (C18:1 *t* plus C18:2 *t*). If necessary, the FA were log-transformed to meet normality requirements. Spearman correlations, linear regression, and paired t-tests were used to compare the liquid and DMS samples. Correlations between liquid and dry milk samples were calculated across the full range of DHA values, and for only those samples with DHA < 1.0 %. The latter is the range in which the vast majority of milk DHA levels lies worldwide [3]. For the stability and inter-lab validation studies, an acceptable range of 15 % of the FA baseline or reference value was determined, as per FDA guidelines [13]. If the FA value remained within the acceptable range over time and in different temperature conditions, it was considered stable. In addition, the entire FA profile was assessed for validity and stability and presented in the Additional files.

## Results

### Validation of liquid vs. DMS FA profile

**Fig. 1:**
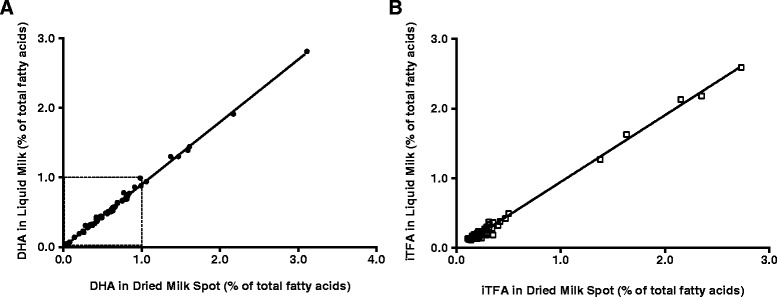
Comparison of liquid with dried milk spot for % DHA (**a**) and %iTFA (**b**; *n* = 55). Regression equations for the whole cohort: Liquid % DHA = 0.8974 (DMS % DHA) – 0.0015 (R^2^ = 0.996, *P* < 0.0001) and Liquid % iTFA = 0.9605 (DMS % iTFA) - 0.0146 (R^2^ = 0.994, *P* < 0.0001). For the subset of samples less than 1 % DHA, the regression equation was: Liquid % DHA = 0.9116 (DMS % DHA) – 0.0054 (R^2^ = 0.981, *P* < 0.0001)

The % DHA of the liquid and DMS samples were highly correlated for both the whole set (R = 0.998, *P* < 0.0001, Fig. 1a) and a subgroup with less than 1 % DHA (R = 0.991, *P* < 0.0001, Fig. 1a). The mean % DHA value was significantly different between the liquid and DMS samples (0.62 ± 0.47 % vs. 0.69 ± 0.52 %, respectively, *P* < 0.0001). The linear regression equation to estimate the liquid milk % DHA using DMS data was: Liquid % DHA = 0.8974 (DMS % DHA) – 0.0015 (R^2^ = 0.996, *P* < 0.0001). For the < 1 % DHA subset, the regression equation was: Liquid % DHA = 0.9116 (DMS % DHA) – 0.0054 (R^2^ = 0.981, *P* < 0.0001).

The % iTFA from the liquid and DMS samples were also highly correlated (R = 0.997, *P* < 0.0001, Fig. 1b). The mean % iTFA value was significantly different between liquid and DMS samples (0.37 ± 0.53 % vs. 0.40 ± 0.55 %, respectively, *P* < 0.0001). The regression equation to estimate liquid % iTFA from DMS was: Liquid % iTFA = 0.9605 (DMS % iTFA) - 0.0146 (R^2^ = 0.994, *P* < 0.0001). There was a greater than 15 % difference between the liquid and DMS samples for six FA (C16:1n7t, C18:1 t, C20:0, C22:0, C24:0, C22:5 n-3; see Additional file 1); however, each of these FA had a mean relative abundance of < 2.5 % of total FA.

### Stability of FA profile in DMS over time at four temperatures

**Fig. 2:**
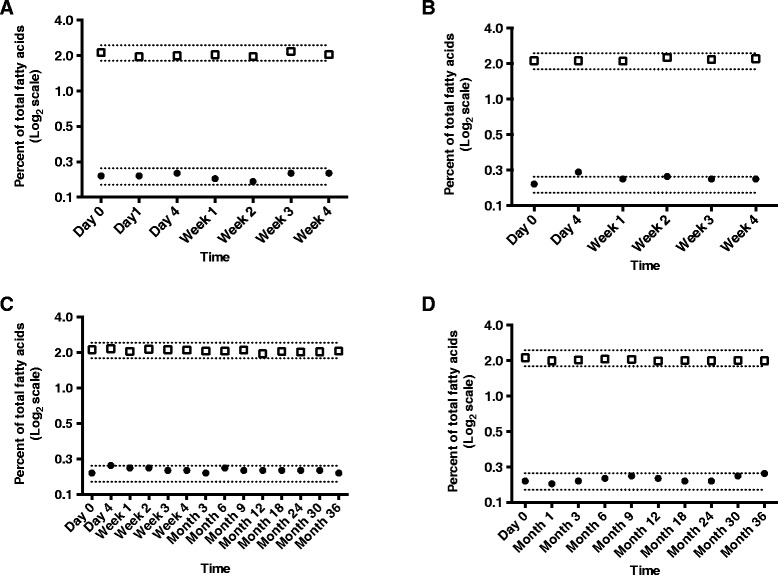
% DHA and % iTFA from dried milk spots (*n* = 5) over time and in different storage conditions. Temperature Storage **a**, 23 °C [room temperature]; **b**, 4 °C [refrigeration]; **c**, -20 °C [standard freezer]; **d**, -80 °C [research freezer]). Dotted lines represent 15 % deviation from baseline values. Open squares represent iTFA values; closed circles represent DHA values.

Compared to day 0, % DHA stayed within the acceptable range at all time points in the room temperature (23 °C) condition for up to 4 weeks or in the -80 °C freezer condition for up to 3 years (Fig. 2). The % DHA increased slightly over the acceptable range at 4 days in both the refrigeration (4 °C) and -20 °C freezer conditions but otherwise remained stable within the acceptable range for four weeks and three years, respectively. In all conditions at all time points % iTFA was stable. Data from all other FA are presented in Additional files 2, 3, 4 and 5.

### Interlab comparison

The % DHA and % iTFA values from five samples analyzed at both OmegaQuant and OmegaQuant–Asia were within 15 % of the referent (Table 1).

**Table 1:** Comparison of % DHA and % iTFA values from identical samples analyzed affiliated labs, OmegaQuant Analytics (Sioux Falls, South Dakota, US) and OmegaQuant-Asia (Seoul, South Korea)

% DHA				
Samples	Reference^a^	Acceptable range^b^	OmegaQuant	OmegaQuant-Asia
1	0.06 %	0.05–0.07 %	0.06 %	0.07 %
2	0.08 %	0.07–0.09 %	0.07 %	0.09 %
3	0.16 %	0.14–0.18 %	0.15 %	0.16 %
4	0.32 %	0.27–0.37 %	0.30 %	0.34 %
5	0.45 %	0.38–0.52 %	0.41 %	0.49 %
Average	0.21 %	0.18–0.25 %	0.20 %	0.23 %
% iTFA				
Samples	Reference^a^	Acceptable range^b^	OmegaQuant	OmegaQuant-Asia
1	2.15 %	1.83–2.48 %	2.22 %	2.09 %
2	2.52 %	2.14–2.89 %	2.65 %	2.38 %
3	2.07 %	1.76–2.38 %	2.23 %	1.92 %
4	1.56 %	1.32–1.79 %	1.69 %	1.42 %
5	1.09 %	0.92–1.25 %	1.10 %	1.08 %
Average	1.88 %	1.60–2.16 %	1.98 %	1.78 %

^a^The reference values for % DHA and % TFA are the average of the values from each lab

^b^Acceptable range is ± 15 % from the reference value

## Discussion

This series of experiments demonstrate that DMS are a valid sample type to test breast milk FA composition, particularly % DHA and % iTFA. The % DHA and % iTFA were highly correlated between the DMS and the liquid milk samples, the current standard for milk collection. Both % DHA and % iTFA were stable at room temperature and -80 °C for up to four weeks and three years, respectively. Finally, affiliated labs using the same methods analyzed identical DMS samples and achieved results within 15 % of the referent. A previous study found that milk FA composition could be measured using a DMS system, but no comparisons with liquid milk or time/temperature stability data were reported [14]. These data suggest that under field research conditions, DMS can be collected, stored and shipped to a laboratory without loss of sample integrity.

Among the many bioactive components in milk [15], fats provide both a source of energy and indispensable building blocks in the form of essential FA, linoleic acid (n-6) and alpha-linolenic acid (n-3). Perhaps more importantly, it also contains the long-chain metabolites arachidonic acid (n-6) and DHA (n-3), which are highly enriched in brain tissue. Likewise, there are unhealthful components that can be carried in milk that can come from diet, such as iTFA [6], or environmental contaminants such as persistent organic pollutants [16]. Unfortunately, DHA is particularly low in the milk of US mothers compared to many other countries (US average 0.20 % vs. worldwide average 0.32 %) [3, 17], and even in the relatively small comparison described here, the five US samples (which were intentionally pooled to span a “wide” range of DHA levels) had a mean DHA level of 0.20 % compared to the 0.74 % in the Malawian women. Similarly, iTFA levels were quite high in women from the US (1.98 %) compared with Malawi (0.24 %). Absent good quality dietary intake data in either cohort, one can only assume that DHA intakes are much higher and iTFA intakes lower in Malawi than in Sioux Falls, SD, US.

Higher DHA levels in milk (and/or corresponding mother and infant blood levels) have been found to be beneficial for neurodevelopment and vision in infants and young children in some studies, but the evidence is not conclusive [1, 2, 18]. Data from Tanzanian tribes with relatively stable lifetime fish intake have found that the mother avoids excessive depletion of her own DHA stores while breastfeeding if her erythrocyte DHA level is ~ 8 % of total FA, which corresponds to a milk DHA level of ~ 1 % [4]. There is not yet enough clinical evidence to recommend “optimal” DHA milk levels for healthy term infants. Premature infants, however, appear to benefit from milk with augmented DHA levels (either from DHA-enriched milk fortifier or mother’s DHA supplementation) because the infant misses out on significant DHA transfer during the third trimester, the period of greatest brain growth and placental DHA transport in pregnancy [19, 20]. Moreover, DHA may decrease the risk of (or help treat) common complications in premature infants, such as bronchopulmonary dysplasia, necrotizing enterocolitis and retinopathy of prematurity [20].

A limitation of this validation study was the use of DMS samples that had been frozen as liquid milk, thawed then spotted onto the filter paper rather than spotted fresh. Due to the timing of the initiation of our collaboration with the research team, it was not possible to do the analyses on fresh samples. However, FA in liquid milk samples stored at -80C are stable and simulating the DMS using thawed milk and storing for 1 week at room temperature still demonstrates the value of DMS compared to traditional collection and storage methods.

Difficulties in collecting, storing and transporting milk samples, particularly in field research, have limited advancements in this area. The method described here had the potential to overcome some of these challenges. One limitation of the DMS method is that (at present), the concentration of FA in milk (e.g., mg/mL) cannot be measured, only the percent composition. However, few researchers in this field would consider this a limitation since the vast majority of milk FA researchers have used the percent composition approach. Its appeal lies in the robustness of the analytical method, the long tradition of use, and the ability to compare results between studies. In addition, since milk percent FA composition is relatively stable during a feed and throughout the day [21, 22], samples may be collected anytime during the day which allows for flexibility in study design. These considerations aside, the primary reason for measuring milk FA concentrations is to determine the total daily FA intake of the infant, and the difficulties associated with this determination are not insignificant. Whereas measuring concentrations per se is not difficult, it is very challenging to measure the other variables needed to calculate the total milk FA intake per day. These include the marked within-day and within-mother variations in both milk volume [23] and milk fat content [24], plus the practical difficulties of precisely determining how much milk the baby actually consumed on a given day. Because of these challenges, it has long been the practice researchers to express milk DHA status as a percent rather than a concentration, which is doable with DMS samples.

## Conclusion

Logistic challenges in collection, storage and transport of samples have impeded studies where simply measuring milk FA composition is the goal. The ability to collect samples as DMS instead of liquid milk and to store and ship at ambient temperature provides a cost-effective, practical alternative without compromising the quality of the FA data. In a parallel setting, dried *blood* spot samples have been used for the determination of blood FA composition for many years [9, 10] and has facilitated FA research [25], particularly in studies of infants and children [26, 27] and in subjects in remote locations [12]. The ability to measure folate, retinol and markers of inborn errors of metabolism (e.g. phenylketonuria) in dried blood spots has revolutionized research and testing in infants [28]. The ability to utilize DMS to measure milk FA (and perhaps other analytes) should facilitate more research on the role of FA nutrition in human development.

### Availability of data and materials

The datasets supporting the conclusions of this article are included in the Additional file 6.

## Additional files

Additional file 1:
**Comparison of fatty acid composition measured in liquid and dried milk spots.** Blue bars, liquid milk means ± 15 %; red bars, dried milk spot FA means. (PDF 63 kb) Additional file 2:
**Fatty acids (C10:0 – C22:6n-3) from dried milk spots stored at room temperature (23C) for 4 weeks.** Error bars represent ± 15 % from baseline values. Fatty acids that represent < 3 % of total fatty acids are in the left panel; all others are in the right panel. Fatty acids with values exceeding 15 % of baseline at any time point: C10:0, C12:0, C20:0, C18:3n-3, C22:0, C20:3n-6, C24:0, C20:5n-3, C24:1n9, C22:4n-6, C22:5n-6, C22:5n-3. (PDF 144 kb) Additional file 3:
**Fatty acids (C10:0 – C22:6n-3) from dried milk spots stored in a refrigerator (4C) for 4 weeks.** Error bars represent ± 15 % from baseline values. Fatty acids that represent < 3 % of total fatty acids are in the left panel; all others are in the right panel. Fatty acids with values exceeding 15 % of baseline at any time point: C16:1n7t, C18:3n-6, C20:3n-6, C18:3n-3, C22:0, C20:4n-6, C24:0, C20:5n-3, C24:1n9, C22:4n-6, C22:5n-6, C22:5n-3, C22:6n-3. (PDF 128 kb) Additional file 4:
**Fatty acids (C10:0 – C22:6n-3) from dried milk spots stored in a standard freezer (-20C) for 3 years.** Error bars represent ± 15 % from baseline values. Fatty acids that represent < 3 % of total fatty acids are in the left panel; all others are in the right panel. Fatty acids with values exceeding 15 % of baseline at any time point: C10:0, C16:1n7t, C20:1n9, C18:3n-3, C20:2n-6, C22:0, C20:3n-6, C20:4n-6, C24:0, C20:5n-3, C24:1n9, C22:4n-6, C22:5n-6, C22:5n-3, C22:6n-3. (PDF 256 kb) Additional file 5:
**Fatty acids (C10:0 – C22:6n-3) from dried milk spots stored in a research-grade freezer (-80C) for 3 years.** Error bars represent ± 15 % from baseline values. Fatty acids that represent < 3 % of total fatty acids are in the left panel; all others are in the right panel. Fatty acids with values exceeding 15 % of baseline at any time point: C10:0, C16:1n7t, C18:3n-6, C20:1n-9, C18:3n-3, C22:0, C20:3n-6, C20:4n-6, C24:0, C20:5n-3, C24:1n-9, C22:4n-6, C22:5n-6, C22:5n-3. (PDF 255 kb) Additional file 6:
**Datasets.** (XLSX 261 kb)

## Abbreviations

DHA: docosahexaenoic acid
DMS: dried milk spot
FA: fatty acids
iTFA: industrial *trans* fatty acids
