# Supplementary figures and images for: Analysis of breast milk fatty acid composition using dried milk samples
Source: Int Breastfeed J. 2016 Jan 25;11:1. doi: 10.1186/s13006-016-0060-2 (PMC4727292; doi:10.1186/s13006-016-0060-2)

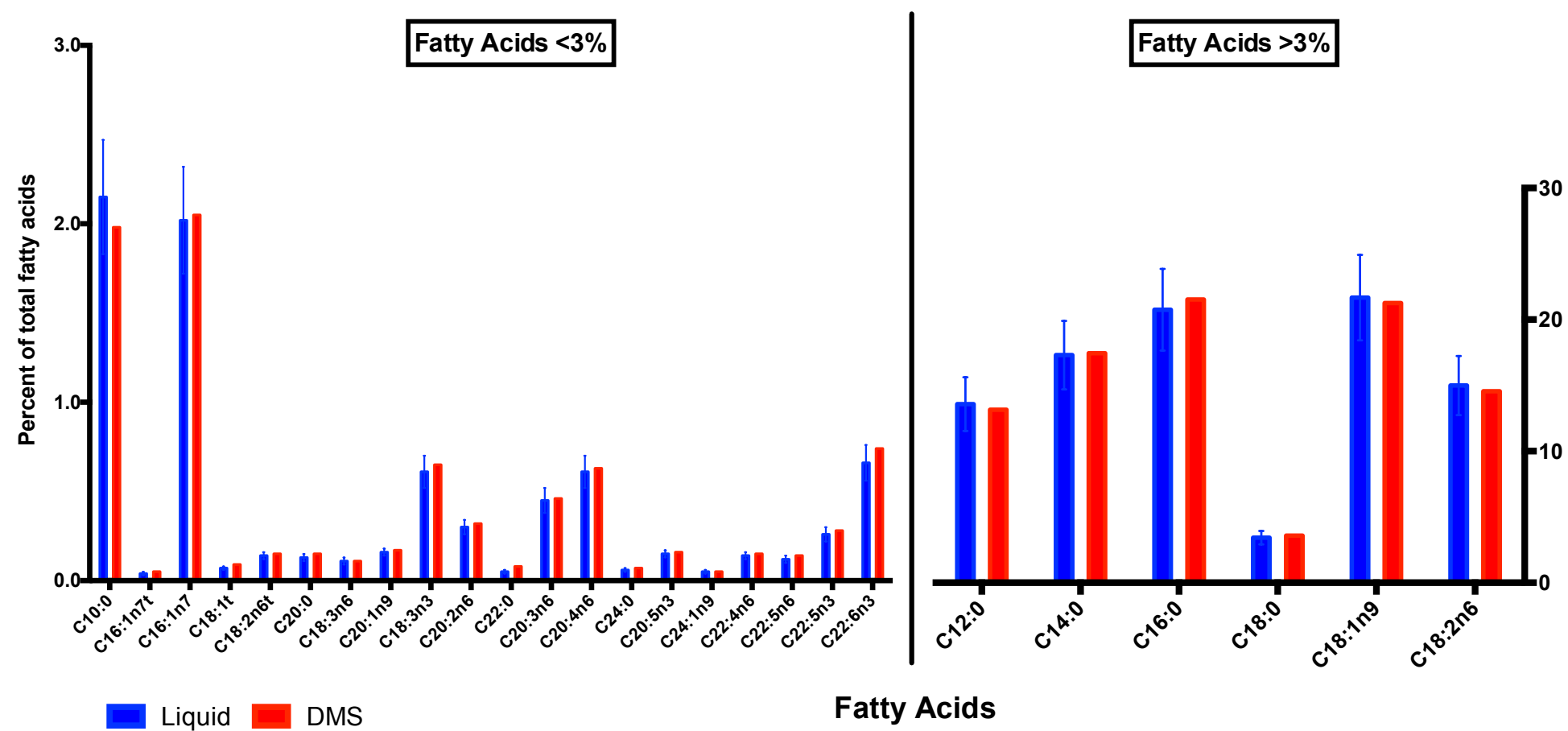

Supplement: Additional file 1: — Comparison of fatty acid composition measured in liquid and dried milk spots. Blue bars, liquid milk means ± 15 %; red bars, dried milk spot FA means. (PDF 63 kb) [file 13006_2016_60_MOESM1_ESM.pdf]

A

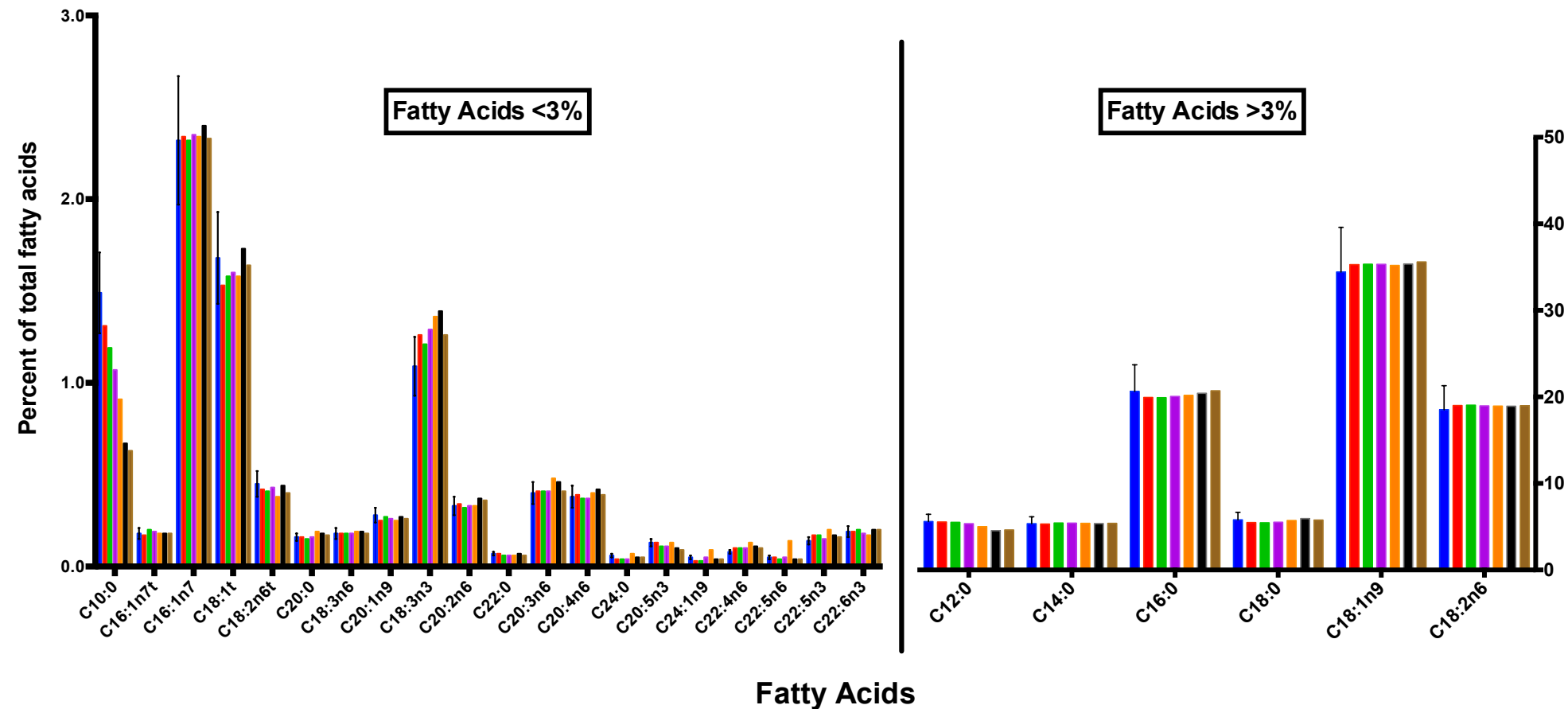

Baseline Day 1 Day 4 Week 1 Week 2 Week 3 Week 4

Supplement: Additional file 2: — Fatty acids (C10:0 – C22:6n-3) from dried milk spots stored at room temperature (23C) for 4 weeks. Error bars represent ± 15 % from baseline values. Fatty acids that represent < 3 % of total fatty acids are in the left panel; all others are in the right panel. Fatty acids with values exceeding 15 % of baseline at any time point: C10:0, C12:0, C20:0, C18:3n-3, C22:0, C20:3n-6, C24:0, C20:5n-3, C24:1n9, C22:4n-6, C22:5n-6, C22:5n-3. (PDF 144 kb) [file 13006_2016_60_MOESM2_ESM.pdf]

**B**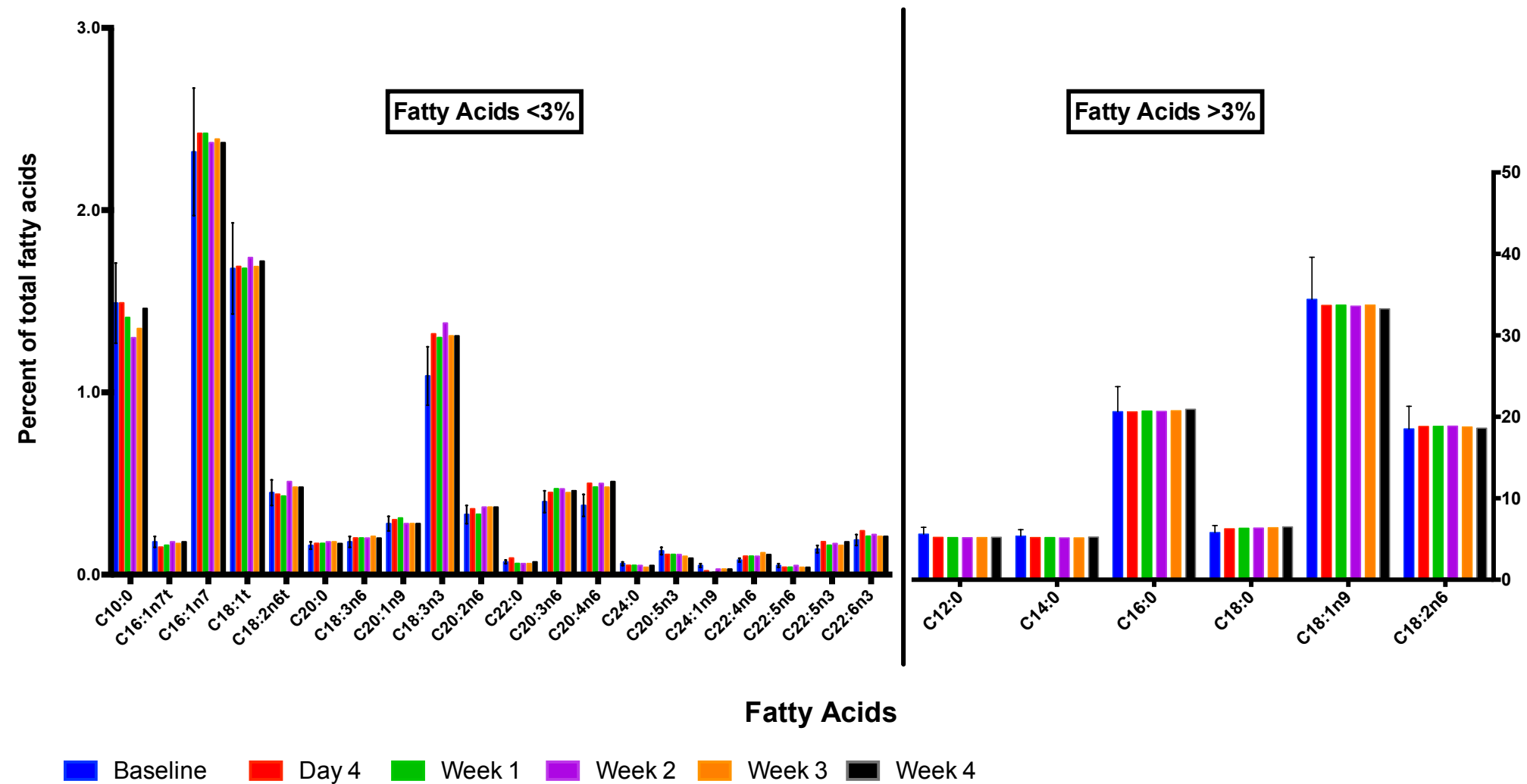

Supplement: Additional file 3: — Fatty acids (C10:0 – C22:6n-3) from dried milk spots stored in a refrigerator (4C) for 4 weeks. Error bars represent ± 15 % from baseline values. Fatty acids that represent < 3 % of total fatty acids are in the left panel; all others are in the right panel. Fatty acids with values exceeding 15 % of baseline at any time point: C16:1n7t, C18:3n-6, C20:3n-6, C18:3n-3, C22:0, C20:4n-6, C24:0, C20:5n-3, C24:1n9, C22:4n-6, C22:5n-6, C22:5n-3, C22:6n-3. (PDF 128 kb) [file 13006_2016_60_MOESM3_ESM.pdf]

D

Fatty Acids &lt;3%

Fatty Acids &gt;3%

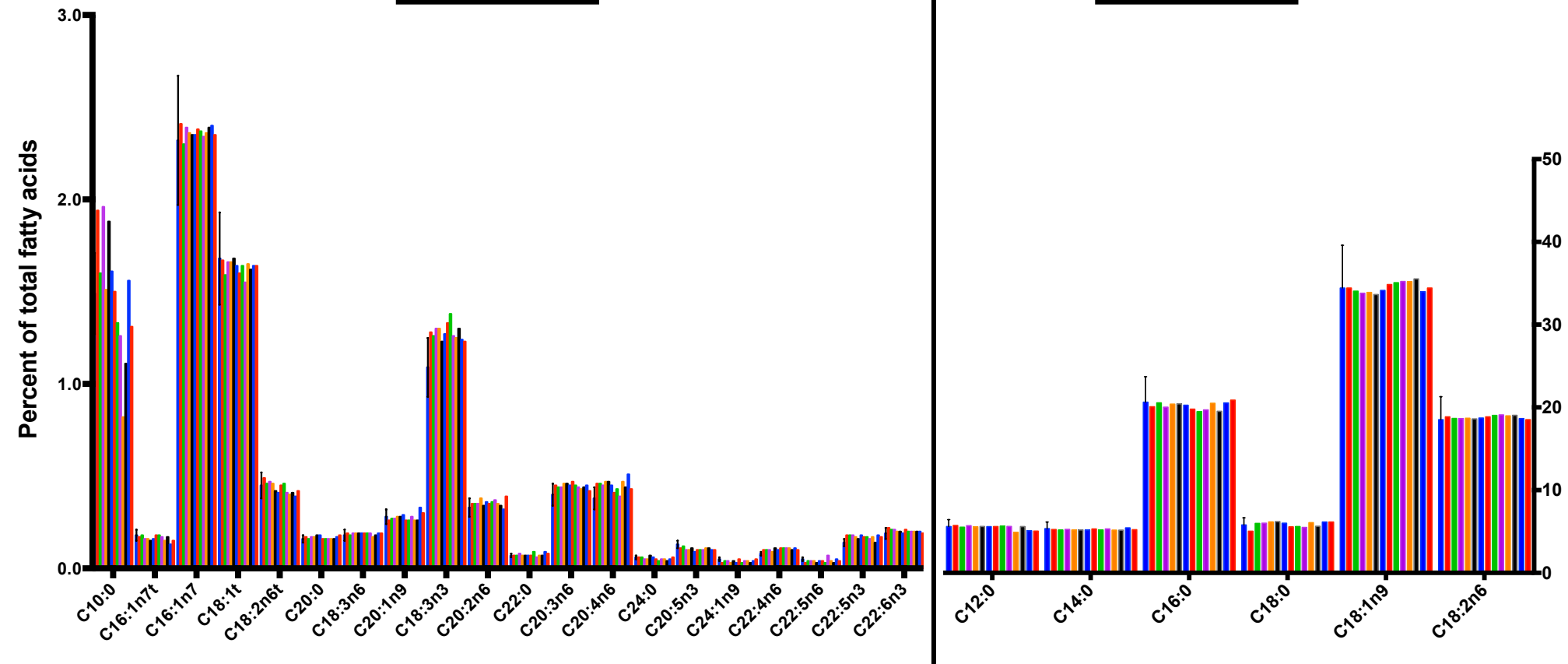

Supplement: Additional file 5: — Fatty acids (C10:0 – C22:6n-3) from dried milk spots stored in a research-grade freezer (-80C) for 3 years. Error bars represent ± 15 % from baseline values. Fatty acids that represent < 3 % of total fatty acids are in the left panel; all others are in the right panel. Fatty acids with values exceeding 15 % of baseline at any time point: C10:0, C16:1n7t, C18:3n-6, C20:1n-9, C18:3n-3, C22:0, C20:3n-6, C20:4n-6, C24:0, C20:5n-3, C24:1n-9, C22:4n-6, C22:5n-6, C22:5n-3. (PDF 255 kb) [file 13006_2016_60_MOESM5_ESM.pdf]
