# Supplementary material for: Analysis of breast milk fatty acid composition using dried milk samples
Source: Int Breastfeed J. 2016 Jan 25;11:1. doi: 10.1186/s13006-016-0060-2 (PMC4727292; doi:10.1186/s13006-016-0060-2)
Supplement: Additional file 4: — Fatty acids (C10:0 – C22:6n-3) from dried milk spots stored in a standard freezer (-20C) for 3 years. Error bars represent ± 15 % from baseline values. Fatty acids that represent < 3 % of total fatty acids are in the left panel; all others are in the right panel. Fatty acids with values exceeding 15 % of baseline at any time point: C10:0, C16:1n7t, C20:1n9, C18:3n-3, C20:2n-6, C22:0, C20:3n-6, C20:4n-6, C24:0, C20:5n-3, C24:1n9, C22:4n-6, C22:5n-6, C22:5n-3, C22:6n-3. (PDF 256 kb) [file 13006_2016_60_MOESM4_ESM.pdf]

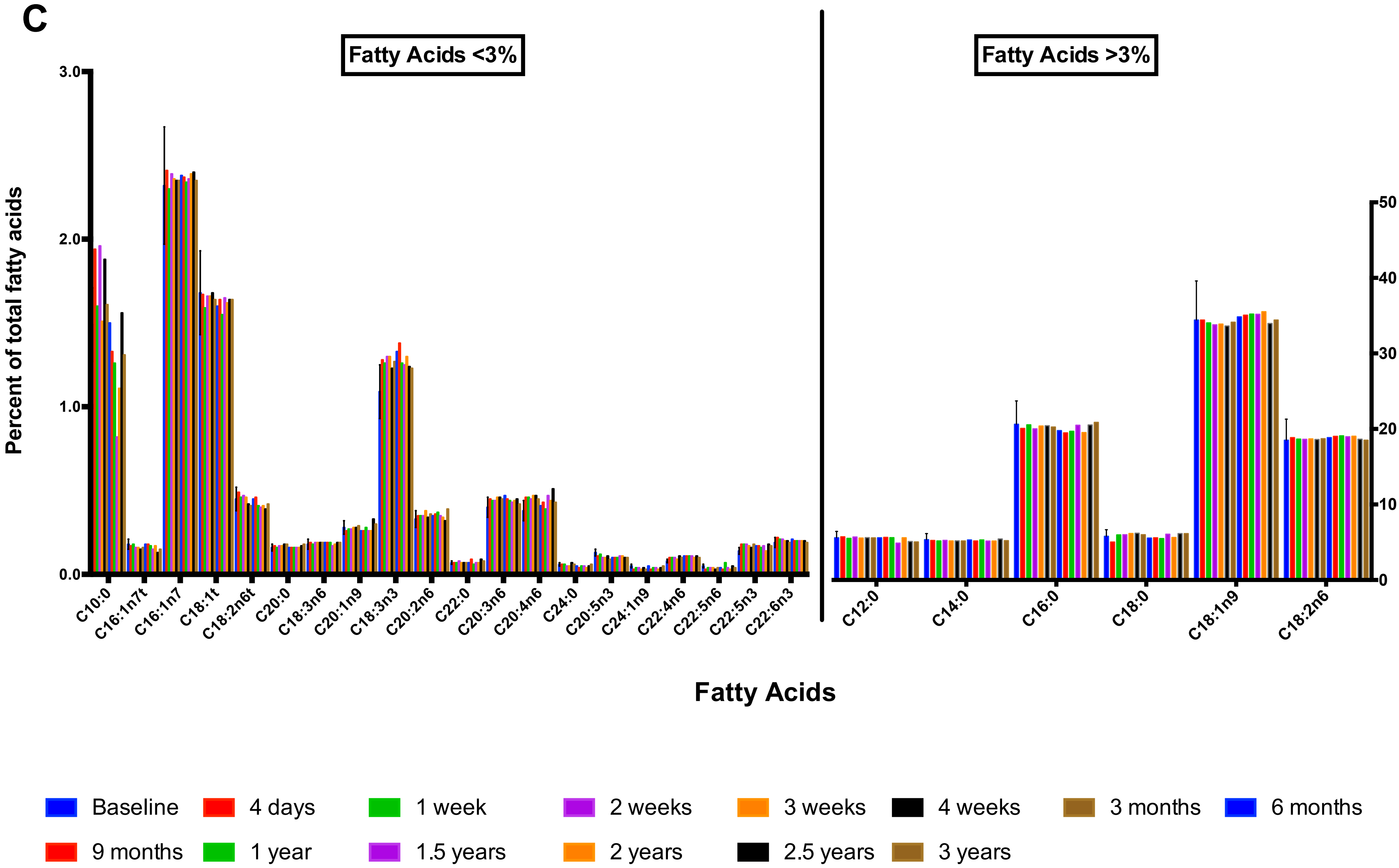
C
Fatty Acids <3%
Fatty Acids >3%
Percent of total fatty acids
3.0
2.0
1.0
0.0
50
40
30
20
10
0
C10:0
C16:1n7t
C16:1n7
C18:1t
C18:2n6t
C20:0
C18:3n6
C20:1n9
C18:3n3
C20:2n6
C22:0
C20:3n6
C20:4n6
C24:0
C20:5n3
C24:1n9
C22:4n6
C22:5n6
C22:5n3
C22:6n3
C12:0
C14:0
C16:0
C18:0
C18:1n9
C18:2n6
Fatty Acids
Baseline
4 days
1 week
2 weeks
3 weeks
4 weeks
3 months
6 months
9 months
1 year
1.5 years
2 years
2.5 years
3 years
